# Evaluating the Net Energy Requirements for Maintenance Based on Indirect Calorimetry and Heart Rate Monitoring in Gestating Sows

**DOI:** 10.3390/ani14192907

**Published:** 2024-10-09

**Authors:** Zhe Li, Wenjun Gao, Huangwei Shi, Song Xu, Zhengcheng Zeng, Fenglai Wang, Changhua Lai, Shuai Zhang

**Affiliations:** 1 State Key Laboratory of Animal Nutrition and Feeding, College of Animal Science and Technology, China Agricultural University, Beijing 100193, China; sjzkyle@163.com (Z.L.); wjgao1119@163.com (W.G.); lzhe47336@gmail.com (H.S.); mprki89@163.com (S.X.); wjgao1119@gmail.com (Z.Z.); wangfl@cau.edu.cn (F.W.); 2 National Center of Technology Innovation for Pigs (North China Branch), Ministry of Agriculture and Rural Affairs Feed Industry Center, China Agricultural University, Beijing 100193, China

**Keywords:** sow, gestation, net energy requirements for maintenance, heart rate, heat production, indirect calorimetry

## Abstract

**Simple Summary:**

This study was conducted to ascertain the net energy requirements for maintenance in gestating sows utilizing indirect calorimetry, and to evaluate the potential of daily heart rate monitoring as a predictive method for these energy needs. In the first experiment, six sows were subjected to varying energy intake levels, and their heat production was measured to determine energy requirements. In the second experiment, heart rate data were collected to explore its predictive value. The results indicated that the net energy requirements for maintenance averaged 410 kJ/BW^0.75^·d^−1^ during late gestation, and it was also found that heart rate monitoring could accurately predict energy requirements, offering a practical tool for managing sow nutrition. These insights are instrumental for refining feeding strategies within pig production, leading to enhanced animal welfare and improved economic efficiency.

**Abstract:**

The objectives of this study were (1) to determine the net energy requirements for the maintenance of gestating sows based on indirect calorimetry, and (2) to explore the feasibility of predicting the net energy requirements for the maintenance of gestating sows based on daily heart rate monitoring. In Exp. 1, six Landrace × Yorkshire crossbred reproductive sows with an initial body weight of 229.5 ± 14.9 kg at d 56 of gestation were randomly assigned to six diverse energy feeding levels using a 6 × 6 Latin square design. The experimental diet was formulated using corn, soybean meal, and wheat bran as major ingredients, and the six feeding levels were set as 1.2, 1.4, 1.6, 1.8, 2.0, and 2.2 times metabolizable energy for maintenance (100 kcal ME/kg BW^0.75^·d^−1^), respectively. The animal trial lasted for six periods with 9 days per period, encompassing 5 days of adaptation, 3 days of calorimetry in fed state, and 1 day of calorimetry in fasting state. In Exp. 2, six Landrace × Yorkshire crossbred pregnant sows with an initial body weight of 232.5 ± 12.5 kg at d 64 were fed a corn–soybean meal diet. All sows were tested in a respiratory calorimetry chamber for a 4 day calorimetry test. The heat production of the gestation sows was measured every 5 min using indirect calorimetry, and the heart rate of the gestating sows was recorded every minute using a belt-shape monitor. The results showed that the net energy requirements for the maintenance of gestating sows significant increased as the gestational stage progressed (*p* < 0.05), and a linear regression model revealed the average net energy requirement for the maintenance of gestating sows was 410 kJ/BW^0.75^ d^−1^ during late gestation (days 70–110). Moreover, the average heart rate of the gestating sows was 84 bpm, and the mathematical model developed to predict the net energy requirements for the maintenance of gestating sows was ${NE}_{m}(kcal/h)=\frac{1990}{1+exp[\frac{136-HR(bpm)}{43}]}$. In conclusion, the average net energy requirement for the maintenance of sows during late gestation was 410 kJ/BW^0.75^ d^−1^, and the utilization of the heart rate monitoring method was found to provide a relevant, accurate prediction for the net energy requirements of sows.

## 1. Introduction

The net energy (NE) system is widely acknowledged as the most precise measure of energy available to pigs [1,2]. The NE system effectively matches the energy requirements for pigs with the amounts of dietary energy on a same basis, regardless of the characteristics of their feed [3]. Indirect calorimetry (IC) has chronically been regarded as the gold standard for measuring the net energy consumed by pigs; numerous studies have employed this method to investigate the energy metabolism of growing pigs, gestating sows, and group-housed pigs [4,5,6]. Furthermore, the detrimental impact of a cramped environments with restricted space and elevated humidity on the welfare of sow animals should not be overlooked. Hence, it is imperative to conduct studies on the energy metabolism of sows using approaches that prioritize their well-being and minimize stress levels [1].

The net energy requirements for maintenance (NE_m_) are presumed to represent the basal energy expenditure (EE) required for basic physiological functions [7,8], and NE_m_ is usually calculated as the heat production (HP) value at which energy intake equals zero [9,10]. Previous studies have reported significant changes in energy utilization in sows during late gestation, especially for the mobilization of body lipids to provide energy to ensure gestational progression [11,12,13]. Unfortunately, there are currently limited data to quantify the NE_m_ of late gestating sows [14,15].

The heart rate (HR) monitoring method is an economical and non-invasive technique developed for the rapid estimation of EE in animals [16]. Because mammals transport O_2_ to the heart, there exists a relationship between HR and EE that can be exploited for EE prediction based on HR [17]. This method has been applied extensively in many species, including humans and free-ranging animals [18,19], but its application on pigs has rarely been reported [20]. Considering the traditional indirect calorimetry method for HP measurement is complex and expensive, the HR method is a more practical alternation that has great potential on the quantitative evaluation of energy metabolism in pigs.

Therefore, this study aims to determine the NE_m_ of sows in the late gestational stage based on indirect calorimetry, and to explore the feasibility of predicting the NE_m_ of gestating sows based on daily heart rate monitoring.

## 2. Materials and Methods

All experimental procedures were approved by the Institutional Animal Care and Use Committee at China Agricultural University (Aw90904202-1-4).

### 2.1. Animals and Diets

Six gestating sows (Landrace × Large White) with an average parity of 5.8 were used in this experiment. At day 55 of gestation (with an average body weight of 229.5 ± 14.9 kg), all sows were transferred into metabolism cages and accommodated on a slatted floor. All sows were manually weighed at the beginning and at the end of the animal trial, were treated with a routine immunization procedure, and had free access to water throughout the experimental period [21].

The experimental diets (in meal form) were formulated using corn, soybean meal, and wheat bran as the major ingredients, and contain 13.4 MJ metabolizable energy (ME)/kg and 15.3% crude protein (Table 1). Sows were fed twice a day at 08:00 and 16:00. To eliminate any impact on pregnancy, feeding allowances were adjusted to keep equivalent energy intake levels for each sow. Six feeding levels, denoted as 1.2 M, 1.4 M, 1.6 M, 1.8 M, 2.0 M, and 2.2 M, were set as 1.2, 1.4, 1.6, 1.8, 2.0, and 2.2 times ME for maintenance (100 kcal ME/kg BW^0.75^·d^−1^), respectively, in compliance with the NRC (2012) guidelines.

### 2.2. Measurement and Sample Collection

The animal trial was designed as a 6 × 6 Latin square design encompassing 6 feeding levels and 6 periods. Within each period, data were collected from 6 sows allotted to the 6 feeding levels kept in 6 respiration chambers. Each period lasted for 9 days, including a 5-day acclimation window for sows to adjust to the experimental diets before moving on to the balance trial, a 3-day calorimetric period for sows staying in fed state, and a 1-day period for sows in fasting state to collect data of fasting heat production (FHP). Before entering into the respiration chambers, all gestating sows were equipped with equipment to continuously monitor their heart rates during calorimetric measurement.

Fecal samples were collected from each sow during meal times twice daily throughout the regular calorimetric period, following the methodologies reported in a previous study [22], and were subsequently stored at −20 °C. Urine was collected into buckets with 10 mL of 6 N HCL being added for every 1000 mL of urine, and 3% (*w*/*w*) of the collected urine was stored at −20 °C. During the fasting period, only urine samples were collected. All fecal samples collected during day 6 to day 8 were homogenized for each sow and subsampled for proximate analysis. Urine samples from each sow were thawed, mixed, and then packaged. Subsequently, subsamples were then taken and pooled for analysis.

### 2.3. Experimental Device

The open-circuit respiration chamber was created using a design similar to that facilities of INRA, with specifications detailed by Li et al. [22]. To facilitate gas exchange, a negative-pressure pulling system was employed, and the gas extraction rate was measured using an Alicat mass flow device (Alicat, Tucson, AZ, USA). Aliquot samples were continuously extracted by a vacuum pump and analyzed for air composition. Gas concentrations were recorded at 5 min intervals, and the gas exchange during regular calorimetry was recorded continuously over a 22 h period. The total heat production of the 22 h gas exchange measurement was then extrapolated to a 24 h value, while fasting heat production was predicted from 8 h heat production from day 9 to day 10 (22:00 to 06:00 h) [23]. The O_2_ concentration was measured using a paramagnetic differential analyzer (Oxymat 6E, Siemens, Munich, Germany), while CO_2_ and CH_4_ concentrations were measured with an infrared analyzer (Ultramat 6E, Siemens, Munich, Germany). All gas analyzers were calibrated before each experimental period to ensure a measurement range of 19.5–21% for O_2_, 0–1% for CO_2_, and 0–0.1% for CH_4_, with a sensitivity of 0.2%. The environment in the respiration chambers was maintained at a constant temperature of 19 °C, a relative humidity of 70%, and a 12 h light–dark cycle from 06:00 to 18:00.

The experiment employed a Polar H10 heart rate monitor (Polar Electro Oy, Kempele, Finland), which consists of an electrode belt, a heart rate sensor, and a smart phone receiver. Similar in principle to an electrocardiogram (ECG), the electrode belt was positioned around the thorax and caudal of the forelimbs’ armpit in the sow, and was moisturized to facilitate optimal contact between the electrode surface and the clean skin surface. The location of the electrode belt was calibrated and moistened after each feeding. Consecutive data were recorded every second by the heart rate sensor, which also had a memory function. The receiver, accessible through a mobile phone application, was connected to the heart rate sensor via Bluetooth^®^ technology. Following data collection, the heart rate records of each sow were downloadable from the Polar Interface.

### 2.4. Chemical Analysis and Calculations

Diet and fecal samples were analyzed for dry matter (DM) (Method 934.01; AOAC, 2007), crude protein (CP) (Method 990.03; AOAC, 2007), ash (Method 942.15; AOAC, 2007), neutral detergent fiber (NDF), and acid detergent fiber (ADF) (Van soest) [24]. Organic matter (OM) was calculated as the difference between DM and ash. Diet samples were also analyzed for ether extract [25]. The gross energy (GE) content in the diet, feces, and urine samples were analyzed using an adiabatic bomb calorimeter (Parr 6300 Calorimeter, Moline, IL, USA).

The apparent total tract digestibility (ATTD) of energy and nutrients was calculated using the direct method [26]. The digestible energy (DE) of diets was calculated as the GE in diets minus the gross energy losses in feces. The metabolizable energy (ME) of diets was calculated as DE minus the gross energy losses in urine and methane. The energy loss in methane (CH_4_E) was calculated using the index of 39.54 kJ per liter of methane emission [27]. The retained energy (RE) was calculated as ME minus gross energy losses as heat. The NE of the diets was calculated as the sum of RE and FHP estimated during the fasting state. The total heat production (THP) was calculated as the average HP during day 6 to day 8 in each calorimetry period. The heat production and respiratory quotient were calculated from gas exchanges following the equations reported by Brouwer [27]:

HP (kJ) = 16.1753 × O_2_ (L) + 5.0208 × CO_2_ (L) − 2.1673 × CH_4_ (L) − 5.9873 × N (Urinary N, g)

RQ = CO_2_ (L/d)/O_2_ (L/d)

Energy retained as protein (RE_p_) was calculated as nitrogen (N) retention (g; difference between N intake and N output in feces and urine) × 6.25 × 23.86 (kJ/g). The energy retained as fat (RE_f_) was calculated as the difference between RE and RE_p_ [28].

### 2.5. Statistical Analysis

To explore the effects of feeding levels and gestation stages on the energy requirements and nutrient digestibility of sows, data were checked for normality to remove outliers using the Distribution procedure in JMP 14.0 (SAS Inst. Inc., Cary, NC, USA) and then were analyzed using the Fit Model procedure in JMP 14.0. The two statistical models included feeding level or gestation stage as the only fixed effect, respectively, and sow and respiration chamber as random effects. The ordinary least square (OLS) algorithm was used to calculate the treatment means, and Tukey’s test was used for multiple comparison to separate treatment means with significant differences. *p* < 0.05 was considered significant. Moreover, linear regression analyses were conducted to determine the relationship between HP or FHP (kJ/kg BW^0.75^·d^–1^) and ME intake (kJ/kg BW^0.75^·d^–1^), and the NE_m_ value was estimated through extrapolating HP at 0 ME intake from the measured HP at 6 gradient feeding levels.

To explore the feasibility of predicting the EE or NE_m_ of gestating sows based on daily heart rate monitoring, the data of the heart rate and EE were displayed in numerical form using Excel 2016. Considering that not all sows monitored gave results of heart rate due to the discontinuous connection of the electrode belt, the effects of the gestation stage and feeding level on the HR or EE were not taken into consideration. The total number of effective HR records was matched with the EE collected at the same time. Two kinds of prediction models were developed: (1) linear models to predict EE based on HR during different daily time slots (05:00 to 21:00, 21:00 to 05:00 next day, and 00:00 to 24:00) were developed and coefficients were estimated based on the OLS algorithm using the lm function in R version 4.2.1, and the R^2^ and RMSE were calculated to evaluate the prediction models; (2) nonlinear mixed-effects models to predict NE_m_ based on HR were developed according to the following logistic mixed models reported previously [29]:

(1) $$g\left(\Phi_{i},{HR}_{ij}\right)=\frac{\phi_{1i}}{1+exp[\frac{\phi_{2i}-{HR}_{ij}}{\phi_{3i}}]}$$

in which *i* represents the sow number and *j* represents the HR data number. The coefficients $\Phi_{i}=\left(\phi_{1i},\;\phi_{2i},\;\phi_{3i}\right)$ were estimated based on the Stochastic Approximation Expectation Maximization (SAEM) algorithm using the saemix package in R version 4.2.1, and several diagnostic fit plots were displayed, including the plot of the observations versus individual predictions, and the plot of the residuals versus HR and versus individual predictions.

## 3. Results

### 3.1. Nutrient Digestibility and Nitrogen Balance

The effects of feeding level or gestation stage on the nutrient digestibility and N balance of sows are presented in Table 2 and Table 3, respectively. Overall, the body weight of all sows increased as gestation progressed (*p* = 0.002). There were no significant differences observed of gradient feeding levels on nutrient digestibility in the late gestating sows (*p* > 0.05). In contrast, the ATTD of DM, CP, and OM significantly increased during the late gestation period (from gestation day 107) compared to the earlier periods (*p* = 0.014, 0.013, and 0.020, respectively), but the ATTD of GE, NDF and ADF were similar regardless of gestation stages (*p* > 0.05). The N intake increased as gestation progressed (*p* = 0.011), and the minimum fecal N was observed during the late gestation period (from gestation day 107) (*p* = 0.018). The N retention was not affected by gestation stage, while the increased feeding level significantly increased the N intake and N retention of the gestating sows (*p* < 0.01).

### 3.2. Energy Balance

**Figure 1:**
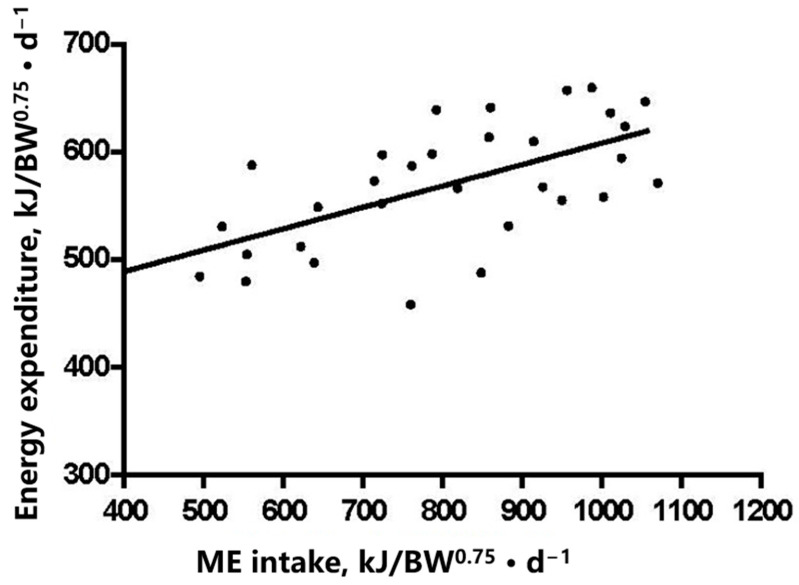
Determination of the net energy requirements for maintenance of sows in late gestation by regression approach with different feeding levels. Linear regression model is HP = 0.199 × ME intake + 409.6 (R^2^ = 0.369, *p* < 0.01).

In the current trial, the average NE_m_ of the sows during late gestation was 410 kJ/kg BW^0.75^·d^−1^, which was calculated based on the linear regression equation illustrated in Figure 1. The effects of feeding levels or gestation stages on the energy balance of sows are presented in Table 4 and Table 5. The FHP increased as gestation progressed (*p* = 0.016), while the sows showed a highest FHP at day 85 after mating, ranging from 387 to 483 kJ/kg BW^0.75^ ·d^−1^. In addition, the FHP occupied 78 and 56% of the THP and ME intake, respectively. The THP, RE, and RE_p_ of the sows increased as feeding level increased, but the FHP was similar among different feeding-level treatments. The respiratory quotient (RQ) of the sows remained unaffected by the gestation stage (*p* > 0.05). However, feeding levels significantly influenced the RQ of sows, with the lowest RQ in the fed state being observed on the 1.2 M feeding group, and then falling below 1.0 when the ME intake level was less than 1.4 M (*p* = 0.02).

### 3.3. Heart Rate

Table 6 shows observations of the daily HR and EE of gestation sows in different time slots in the current study. During the day time slot (05:00~21:00), the HR of sows ranged from 57 to 149 bpm, which was slightly greater than that during the night time slot, with a range of 62 to 134 bpm. The average EE measured in the current study was 477.1 kJ/kg BW^0.75^·d^−1^. Additionally, the correlation coefficient between EE and HP during the day time slot (*r* = 0.74) was greater than that obtained during the night time slot (*r* = 0.60). The linear model, developed for EE prediction based on HR data collected throughout the day in gestating sows, is

EE (kJ/BW^0.75^·d^−1^) = 7.053 HR (bpm) − 117.9 (R^2^ = 0.55, RMSE = 105.5, *p* < 0.01). (2)

**Figure 2:**
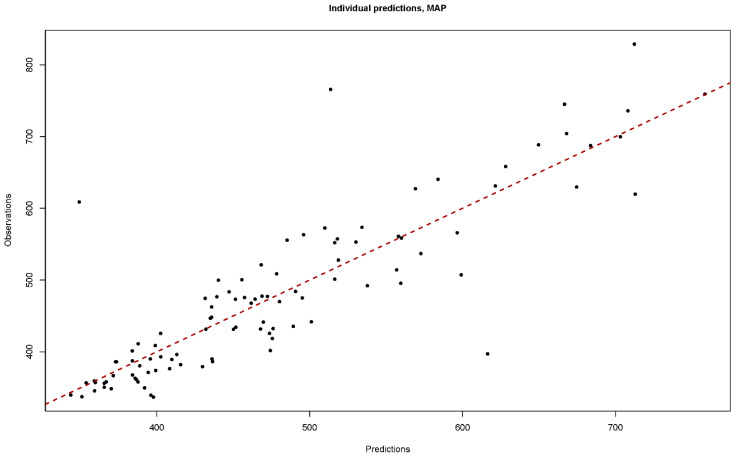
The observations versus individual prediction plot to diagnose the goodness of fit of the nonlinear mixed models to predict the net energy requirements for the maintenance of sows in late gestation based on heart rate monitoring.

**Figure 3:**
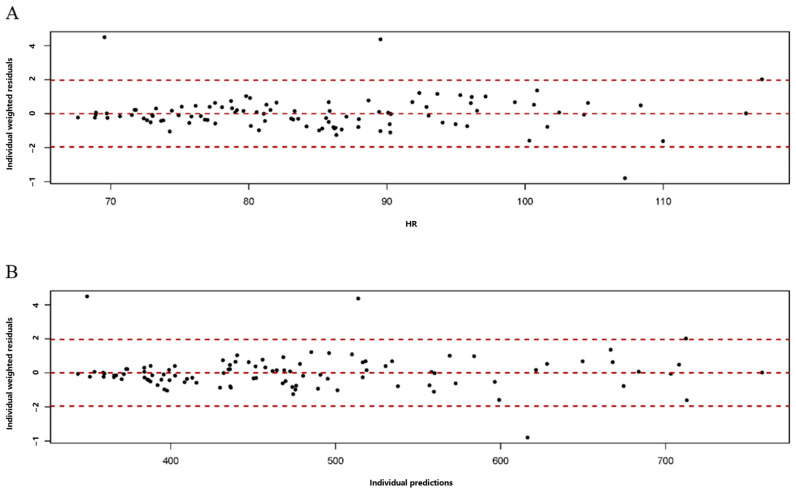
The residuals versus heart rate (HR) observation plot (**A**) and the residuals versus individual prediction plot (**B**) to diagnose the goodness of fit of the nonlinear mixed models to predict the net energy requirements for the maintenance of sows in late gestation based on HR monitoring.

Moreover, the logistic mixed model developed for NE_m_ prediction based on the HR data of gestating sows is

(3) $${NE}_{m}(kcal/h)=\frac{1990}{1+exp[\frac{136-HR(bpm)}{43}]}$$

with the parameter estimation results shown in Table 7. The diagnostic fit plots, including the plot of the observations versus individual predictions (Figure 2) and the plot of the residuals versus HR (Figure 3A) and versus individual predictions (Figure 3B) all demonstrated goodness of fit to the developed nonlinear mixed model.

The model is $g\left(\Phi_{i},{HR}_{ij}\right)=\frac{\phi_{1i}}{1+exp[\frac{\phi_{2i}-{HR}_{ij}}{\phi_{3i}}]}$, and coefficients were estimated using the stochastic approximation expectation maximization algorithm.

## 4. Discussion

The net energy requirement for maintenance is typically thought of as heat dissipated into the environment, which is considered proportional to the metabolic body weight (BW^0.75^), and can be approximately estimated as the fasting heat production [30]. However, abnormal metabolism is usually observed during NE_m_ measurement using the fasting approach [31,32]; thus, an alternative evaluation method is required, such as the regression method, for NE_m_ measurement [9,33]. In the current study, the average NE_m_ during late gestation, extrapolated using the regression method, was approximately 410 kJ/kg BW^0.75^·d^−1^, greater than the values obtained by Ramonet et al. (310 kJ/kg BW^0.75^·d^−1^) [34] and Wang et al. (326 kJ/kg BW^0.75^·d^−1^) [6]. Discrepancies between these values could be attributed to differences in experimental design, animal breeds and conditions, and management practices. For instance, previous studies suggested that the NE_m_ values of growing pigs determined using the regression method were greater than those obtained using the fasting method due to the differences in pig activity between the fasting and fed states [8]. Furthermore, sows with higher parities usually required more energy to maintain gestation and overall health [35], leading to greater NE_m_ compared to lower-parity sows [15,33]. The extreme high parity (averaged 5.8) of the sows used in the current trial may have greatly contributed to the greater NE_m_ measurements. Notably, the sows used in this study received vaccinations to ensure the health of the piglets, which may also introduce variability in NE_m_ determination [36]. In addition, when the ME intake was below 1.4 M, the RQ of the sows was below 1.0, indicating an imbalanced energy status. Therefore, increasing energy intake levels may be required to regulate this imbalance.

In late gestation, the FHP values of the sows were in the range of 387~483 kJ/kg BW^0.75^·d^−1^ in the current study, and were found to be significantly increased with the progression of pregnancy, which was also reported in a previous study [37]. However, the FHP values were not affected by the previous feeding level, contrary to the findings obtained in growing pigs [31]. This phenomenon may be attributed to the differences in digestive tract size between pregnant sows and growing pigs. Protein requirements were reported to increase 19-fold after day 70 of gestation [38], which is in agreement with the findings that most of the BW gain occurs during the late gestation of sows [39]. On the other hand, uterine blood flow rate showed a 2-fold increase from day 44 to day 111 of gestation [40], and the visceral organ mass and associated HP were reported to be significantly increased as pregnancy progressed [11,41], indicating that all the active metabolizable tissues of sows were mobilized to provide the necessary energy required for maintenance and reproduction.

The method of using heart rate to quantify energy expenditure has been widely utilized in both free-ranging animals and humans [17,19], but the correlation coefficients and the corresponding prediction models of the HR method on sows remain underexplored. In the 1980s, Spurr [42] proposed the development of the “flex-HR” method, which sought to address the variability in HR responses among different subjects. The purpose of this methodology was to enhance the comprehension of human energy requirements by establishing personalized calibrations between HR and EE for each subject [19]. Wide variations in HR values resulted from various factors such as the environment, activity, and physiological stage [43]. In this study, the HR records of the sows were between 57 and 149 bpm, which is consistent with previous reports by Marchant-Forde et al. [44]. The current results showed that HR is a good predictor of EE during the day time slot, given its better correlation coefficient compared to the results obtained during the night time slot. It has been demonstrated that the physiological stage and activity significantly affected HR and EE [45]. Furthermore, a linear relationship between HR and HP observed through a doubly labeled water method was established [45]. The higher correlation coefficient during daytime could be caused by vigorous activities, similar to what has been reported in other animals [46]. Due to pregnancy, gestating sows have a preference for sleeping and lying down rather than standing or sitting [47]. Their reduced levels of physical activity could impact the accuracy of HR monitoring. Therefore, we included the Beghin’s method into our calculations to improve the computation of EE using HR data [48]. Moreover, Marchant and Broom [46] demonstrated that the basal heart rate of sows increases as gestation progresses, especially during the last third of gestation. This increase in metabolic rate is well known as a result of special physiological requirements during late gestation [49]. Thus, it can be concluded that there is a positive relationship between the heart rate and metabolic rate in sows. The correlation coefficient (0.72) calculated from the linear regression model showed that the HR method is a reliable predictor of the EE of sows, but this value was found to be less accurate when compared to other animals in controlled laboratory conditions [16]. In order to enhance our understanding of the relationship between HR and NE_m_ in pregnant sows, we investigated the utilization of a nonlinear mixed model that was initially proposed by Kortelainen et al. [29] in studies on HR-EE relationships in human. This model effectively handles outliers in heat production that arise from high HRs, resulting in a more accurate fit for sows during their resting state. Additionally, it overcomes the limitations of linear models when dealing with small effective values, subsequently improving the predictive capability of net energy requirement models. Consequently, further research is necessary to better capture the energy metabolism state in late gestation when employing the HR method on sows.

## 5. Conclusions

The net energy requirement for the maintenance of sows in late gestation estimated from a regression approach was 410 kJ/kg BW^0.75^·d^−1^, and the FHP can be influenced by the gestation stage. The linear and nonlinear models developed in this study to predict the EE and NE_m_ of gestating sows were as follows:

EE (kJ/BW^0.75^·d^−1^) = 7.053 HR (bpm) − 117.9 (R^2^ = 0.5537, RMSE = 105.5, *p* < 0.01), and ${NE}_{m}(kcal/h)=\frac{1990}{1+exp[\frac{136-HR(bpm)}{43}]}$, respectively. This enables the application of the HR method on sows in late gestation.

## Figures and Tables

**Table 1:** Nutrient compositions of the experimental diet (%, as-fed basis).

Ingredients	Content
Corn	60.90
Soybean meal	18.00
Wheat bran	16.00
Soybean oil	2.00
Dicalcium phosphate	1.20
Limestone	1.10
Salt	0.30
Premix ^1^	0.50
Total	100.00
Nutrient levels ^2^	
GE, MJ/kg	16.50
CP	15.08
EE	3.93
Starch	41.60
NDF	26.26
ADF	5.40
Ash	4.73
Ca	0.82
P	0.66

^1^ Premix provided per kilogram of complete feed: 6000 IU of vitamin A, 3000 IU of vitamin D3, 20 IU of vitamin E, 1.8 mg of vitamin K3, 2.0 mg of vitamin B1, 6.0 mg of vitamin B2, 4.0 mg of vitamin B6, 3000 mg of choline, 0.02 mg of vitamin B12, 26.0 mg of niacin, 18.0 mg of pantothenic acid, 3.2 mg of folic acid, 0.4 mg of biotin, 120 mg of Fe, 20 mg of Cu, 100 mg of Zn, 50 mg of Mn, 1.2 mg of I, 0.30 mg of Se, 8.0 g of Ca, 0.8 g of P, 5.6 g of NaCl, and 0.05% of lysine. ^2^ GE means gross energy, CP means crude protein, EE means ether extract, NDF means neutral detergent fiber, and ADF means acid detergent fiber.

**Table 2:** Effects of gestation stages on nutrient digestibility and nitrogen balance in gestating sows.

Items ^1^	Days of Gestation, d						SEM	*p*-Value
	67	75	83	91	99	107		
BW, kg	231.9 ^c^	237.1 ^bc^	243.8 ^abc^	250.5 ^abc^	256.7 ^ab^	263.2 ^a^	5.2	0.002
DM intake, kg/d	3.0	3.1	3.1	3.2	3.3	3.3	0.3	0.98
Digestibility coefficients, %								
DM	84.9 ^ab^	85.7 ^ab^	85.9 ^ab^	85.7 ^ab^	82.6 ^b^	88.7 ^a^	0.9	0.014
GE	85.6	86.3	86.6	86.0	85.9	90.7	1.6	0.24
CP	86.5 ^ab^	87.2 ^ab^	87.5 ^ab^	87.2 ^ab^	84.3 ^b^	90.2 ^a^	0.9	0.013
NDF	77.0	79.0	79.7	81.9	73.5	80.8	2.3	0.26
ADF	63.0	66.7	66.3	70.0	56.0	69.6	3.9	0.25
OM	87.7 ^ab^	88.3 ^ab^	88.7 ^ab^	88.1 ^ab^	85.4 ^b^	90.4 ^a^	0.8	0.020
Nitrogen balance, g/d								
N Intake	75.2 ^b^	76.2 ^ab^	77.3 ^ab^	80.0 ^ab^	81.0 ^ab^	82.5 ^a^	1.5	0.011
Fecal excretion	10.2 ^ab^	9.7 ^ab^	9.5 ^ab^	10.1 ^ab^	12.1 ^a^	8.6 ^b^	0.7	0.018
Urine excretion	34.0	37.6	23.3	28.6	23.5	25.7	6.1	0.52
N Retention	31.0	28.9	44.5	41.3	41.2	53.1	6.0	0.17

^a–c^ Means within a row with different superscripts differ (*p* < 0.05). ^1^ BW means body weight, DM means dry matter, GE means gross energy, CP means crude protein, NDF means neutral detergent fiber, ADF means acid detergent fiber, and OM means organic matter.

**Table 3:** Effects of dietary feeding levels on nutrient digestibility and nitrogen balance in gestating sows.

Items ^1^	Energy Allowance Levels						SEM	*p*-Value
	1.2 M	1.4 M	1.6 M	1.8 M	2.0 M	2.2 M		
BW, kg	245.6	246.6	246.4	247.3	250.5	246.8	5.6	0.99
DM intake, kg/d	2.2 ^a^	2.6 ^b^	3.0 ^c^	3.4 ^d^	3.8 ^e^	4.1 ^f^	0.6	<0.01
Digestibility coefficients, %								
DM	84.7	85.0	85.5	85.7	86.9	85.6	0.9	0.95
GE	87.8	85.7	86.1	86.1	89.5	86.0	1.6	0.50
CP	87.3	86.5	86.8	87.1	88.2	87.4	0.9	0.89
NDF	77.3	77.3	78.5	78.8	80.3	80.3	2.3	0.95
ADF	62.8	63.3	65.8	64.9	68.3	67.5	3.9	0.25
OM	87.5	87.7	88.3	88.1	89.2	88.0	0.8	0.97
Nitrogen balance, g/d								
N Intake	55.1 ^f^	64.6 ^e^	74.0 ^d^	83.6 ^c^	93.8 ^b^	101.2 ^a^	1.5	<0.01
Fecal excretion	6.9 ^c^	8.7 ^bc^	9.8 ^ab^	10.7 ^ab^	10.8 ^ab^	12.7 ^a^	0.7	<0.01
Urine excretion	25.0	33.0	21.6	30.2	38.0	27.2	6.1	0.56
N Retention	22.6 ^b^	22.9 ^b^	42.6 ^ab^	42.6 ^ab^	43.3 ^ab^	61.2 ^a^	6.0	<0.01

^a–f^ Means within a row with different superscripts differ (*p* < 0.05). ^1^ BW means body weight, DM means dry matter, GE means gross energy, CP means crude protein, NDF means neutral detergent fiber, ADF means acid detergent fiber, and OM means organic matter.

**Table 4:** Effects of gestation stages on energy balance and respiratory quotient in gestating sows.

Items ^1^	Days of Gestation, d						SEM	*p*-Value
	67	75	83	91	99	107		
No. of sows	6	6	6	6	6	6		
Energy balance, kJ/kg BW^0.75^/d								
ME intake, MEI	785.2 ^ab^	790.4 ^ab^	799.3 ^ab^	790.7 ^ab^	735.3 ^b^	883.1 ^a^	10.42	0.87
Total heat production, THP	530.1	569.7	611.1	566.0	574.9	579.7	21.08	0.17
Fasting heat production, FHP	386.9 ^b^	421.6 ^ab^	482.5 ^a^	478.3 ^a^	466.2 ^ab^	453.9 ^ab^	21.63	0.02
Retained Energy, kJ/kg BW^0.75^/d								
As protein, RE_p_	77.6	71.3	108.5	97.1	97.4	121.3	14.08	0.28
As fat, RE_f_	177.5	104.4	67.3	127.6	63.0	182.1	29.41	0.14
Retained energy, RE	255.1	175.7	175.8	224.6	133.7	252.8	34.81	0.12
Energy utilization, %								
UE:DE	4.7	5.2	3.2	4.0	4.0	1.6	0.00	0.31
CH_4_E:DE	0.5	0.6	0.8	0.9	0.8	0.6	0.11	0.18
ME:DE	94.8	94.4	96.0	95.1	95.2	97.9	0.90	0.30
NE:ME	82.0	81.9	82.8	88.3	85.9	86.3	2.12	0.17
Respiratory quotient, RQ								
Fed state	0.97	0.99	1.02	1.00	1.00	1.00	0.16	0.26
Fasted state	0.77	0.81	0.83	0.69	0.70	0.71	0.10	0.87

^a,b^ Means within a row with different superscripts differ (*p* < 0.05). ^1^ ME means metabolizable energy, UE means urinary energy, CH_4_E means methane energy, and NE means net energy.

**Table 5:** Effects of dietary feeding levels on energy balance and respiratory quotient in gestating sows.

Items ^1^	Energy Allowance Levels						SEM	*p*-Value
	1.2 M	1.4 M	1.6 M	1.8 M	2.0 M	2.2 M		
No. of sows	6	6	6	6	6	6	6	6
Energy balance, kJ/kg BW^0.75^/d								
ME intake, MEI	537.9 ^f^	650.3 ^e^	752.1 ^d^	537.9 ^f^	650.3 ^e^	752.1 ^d^	10.38	<0.01
Total heat production, THP	517.9 ^b^	533.1 ^ab^	567.2 ^ab^	517.9 ^b^	533.1 ^ab^	567.2 ^ab^	21.07	<0.05
Fasting heat production, FHP	420.6	460.9	439.0	420.6	460.9	439.0	21.61	0.56
Retained Energy, kJ/kg BW^0.75^/d								
As protein, RE_p_	54.4 ^b^	55.6 ^b^	102.2 ^ab^	54.4 ^b^	55.6 ^b^	102.2 ^ab^	14.12	<0.01
As fat, RE_f_	−34.5 ^b^	15.3 ^b^	52.3 ^bc^	−34.5 ^b^	15.3 ^b^	52.3 ^bc^	29.38	<0.01
Retained energy, RE	16.6 ^c^	70.8 ^c^	154.5 ^bc^	16.6 ^c^	70.8 ^c^	154.5 ^bc^	34.82	<0.01
Energy utilization, %								
UE:DE	6.4	3.7	3.1	6.4	3.7	3.1	0.02	<0.05
CH_4_E:DE	0.8	0.7	0.8	0.8	0.7	0.8	0.11	0.76
ME:DE	92.9	95.7	96.2	92.9	95.7	96.2	10.38	<0.01
NE:ME	82.7	70.5	69.6	82.7	70.5	69.6	21.07	<0.05
Respiratory quotient, RQ							21.61	0.56
Fed state	0.95 ^b^	0.97 ^ab^	1.00 ^ab^	0.95 ^b^	0.97 ^ab^	1.00 ^ab^		
Fasted state	0.68	0.82	0.82	0.68	0.82	0.82	14.12	<0.01

^a–f^ Means within a row with different superscripts differ (*p* < 0.05) ^1^ ME means metabolizable energy, UE means urinary energy, CH_4_E means methane energy, and NE means net energy.

**Table 6:** The average heart rate and energy expenditure records and linear models developed for energy expenditure prediction based on heart rate monitoring in gestating sows.

No. of Sows	Time	Heart Rate, bpm	Energy Expenditure, kJ/BW^0.75^ d^−1^	Correlation Coefficient, *r*	Regression Coefficient, *a* *	Intercept, *b* *	RMSE	R^2^	*p-*Value
6	05:00~21:00	86 (57~149) **	513.6	0.7441	7.6996	−154.27	117.6	0.5537	<0.01
6	21:00~05:00	81 (62~134) **	416.9	0.5974	3.3535	147.03	53.2	0.3569	<0.01
6	00:00~24:00	84 (57~149) **	477.1	0.7211	7.053	−117.88	105.5	0.5200	<0.01

* coefficients in linear model: Y = a + bX, Y = HP (kJ/BW^0.75^ d^−1^), X = HR (bpm). ** heart rate range (min~max).

**Table 7:** The coefficients estimated in logistic mixed model developed for net energy requirement prediction based on heart rate monitoring in gestating sows.

Coefficients	Estimation	Standard Error	CV, %
$\phi_{1}$	1990	936.2	47.0
$\phi_{2}$	136	35.8	26.4
$\phi_{3}$	43	8.0	18.4

## Data Availability

The original contributions presented in the study are included in the article, further inquiries can be directed to the corresponding authors.
